# The role of interleukin-17 in inflammation-related cancers

**DOI:** 10.3389/fimmu.2024.1479505

**Published:** 2025-01-21

**Authors:** Xingru Zhang, Bangjie Li, Tian Lan, Conner Chiari, Xiaoyang Ye, Kepeng Wang, Ju Chen

**Affiliations:** ^1^ The Eighth Clinical Medical College of Guangzhou University of Chinese Medicine, Foshan Hospital of Traditional Chinese Medicine, Foshan, Guangdong, China; ^2^ Department of Pharmacology, School of Life Sciences and Biopharmaceutical Science, Shenyang Pharmaceutical University, Shenyang, China; ^3^ Department of Immunology, School of Medicine, University of Connecticut Health Center, Farmington, CT, United States; ^4^ College of Engineering, Northeastern University, Seattle, WA, United States

**Keywords:** interleukin-17, cancer, inflammation, tumor microenvironment, immunotherapy

## Abstract

Emerging evidence indicates a correlation between inflammation and the development and progression of cancer. Among the various inflammatory signals, interleukin-17 (IL-17) family cytokines serve as a critical link between inflammation and cancer. IL-17 is a highly versatile pro-inflammatory cytokine that plays a pivotal role in host defense, tissue repair, the pathogenesis of inflammatory diseases, and cancer progression. During the early stages of tumorigenesis, IL-17 signaling directly promotes the proliferation of tumor cells. Conversely, IL-17 has been shown to exhibit antitumor immunity in several models of grafted subcutaneous tumors. Additionally, dynamic changes in the microbiome can influence the secretion of IL-17, thereby affecting tumor development. The specific role of IL-17 is contingent upon its functional classification, spatiotemporal characteristics, and the stage of tumor development. In this review, we introduce the fundamental biology of IL-17 and the expression profile of its receptors in cancer, while also reviewing and discussing recent advancements regarding the pleiotropic effects and mechanisms of IL-17 in inflammation-related cancers. Furthermore, we supplement our discussion with insights into the mechanisms by which IL-17 impacts cancer progression through interactions with the microbiota, and we explore the implications of IL-17 in cancer therapy. This comprehensive analysis aims to enhance our understanding of IL-17 and its potential role in cancer treatment.

## 1 Introduction

Interleukin-17 (IL-17), also known as interleukin-17A (IL-17A), was the first identified member of the IL-17 family and has been extensively studied. IL-17 is produced by various immune cell types, including T helper 17 cells (Th17), cytotoxic T cells (Tc17), gamma delta T cells (γδT), natural killer T cells (NKT), and natural killer cells (NK cells), and innate lymphoid cells (1). The IL-17 family consists of six members: IL-17A, IL-17B, IL-17C, IL-17D, IL-17E and IL-17F. Among these IL-17F exhibits the highest homology with IL-17A, while IL-17E shows the least. Notably, IL-17A and IL-17F can form heterodimers, whereas the other IL-17 family cytokines function as homodimers when binding to their respective receptors (2). Currently, the IL-17 receptor (IL-17R) family comprises five members: (IL-17RA, IL-17RB, IL-17RC, IL-17RD, and IL-17RE), all of which are characterized as single-pass transmembrane receptors. Each receptor contains two extracellular fibronectin II-like domains and a cytoplasmatic “SEFIR (SEF/IL-17R)” motif, which is crucial for the activation of downstream signaling pathways (3). Different IL-17 family cytokines originate from distinct cellular sources and are associated with various functions. Specifically, IL-17A, IL-17F, IL-17C, and IL-17E play significant roles in the host defense against pathogens and are implicated in autoimmune, allergic, and chronic inflammatory conditions (4). Given its established role in autoimmunity, IL-17A blocking antibodies, such as secukinumab and ixekizumab, have received U.S. Food and Drug Administration (FDA) approval for the treatment of psoriasis, ankylosing spondylitis (AS), and psoriatic arthritis (5). Elevated expression of IL-17 family cytokines and their receptors has also been observed in various human cancers, including colorectal, ovarian, lung, breast, gastric, skin, hepatic, and head and neck cancers (6). The oncogenic role of IL-17 has been supported by studies focusing on colorectal, breast, lung, and other cancers. These investigations reveal that IL-17 stimulates a diverse array of cytokines, chemokines, and inflammatory mediators, which collectively enhance tumor cell proliferation, migration, and invasion, inhibit apoptosis in tumor cells, undermine anti-tumor responses, promote angiogenesis, and facilitate tumor progression (7). Additionally, IL-17 can promote tumor growth through interactions with other immune cells such as myeloid-derived suppressor cells (MDSCs), neutrophils, and regulatory T cells (Tregs). Conversely, evidence also suggests that IL-17 may enhance or augment the activity of cytotoxic T lymphocytes (CTLs) and natural killer Cells (NK), contributing to anti-tumor effects (8). Furthermore, IL-17 has also been implicated in the influence of the microbiome on cancer progression. In colorectal and lung cancers, the microbiome has been shown to affect tumor progression by regulating the release of IL-17, either directly or indirectly (9). Microbial-dependent IL-17 signaling has been found to enhance DUOX2 signaling in tumor cells associated with pancreatic ductal adenocarcinoma (9). Thus, IL-17 not only influences tumor occurrence and development of tumors but also impacts the efficacy of cancer treatments. Research indicates that IL-17 can increase tumor cell resistance to chemotherapy, induce inflammation, and exacerbate the toxicity of radiotherapy (10). However, some studies suggest that IL-17 may also be protective during radiotherapy (11). Therefore, immunotherapy targeting IL-17 and signaling pathways will provide more treatment options for cancer treatment. In this review, we focus on the role of IL-17 in the Tumor microenvironment (TME) and their therapeutic strategies against cancer.

## 2 Cellular sources of IL-17 in the TME

**Figure 1:**
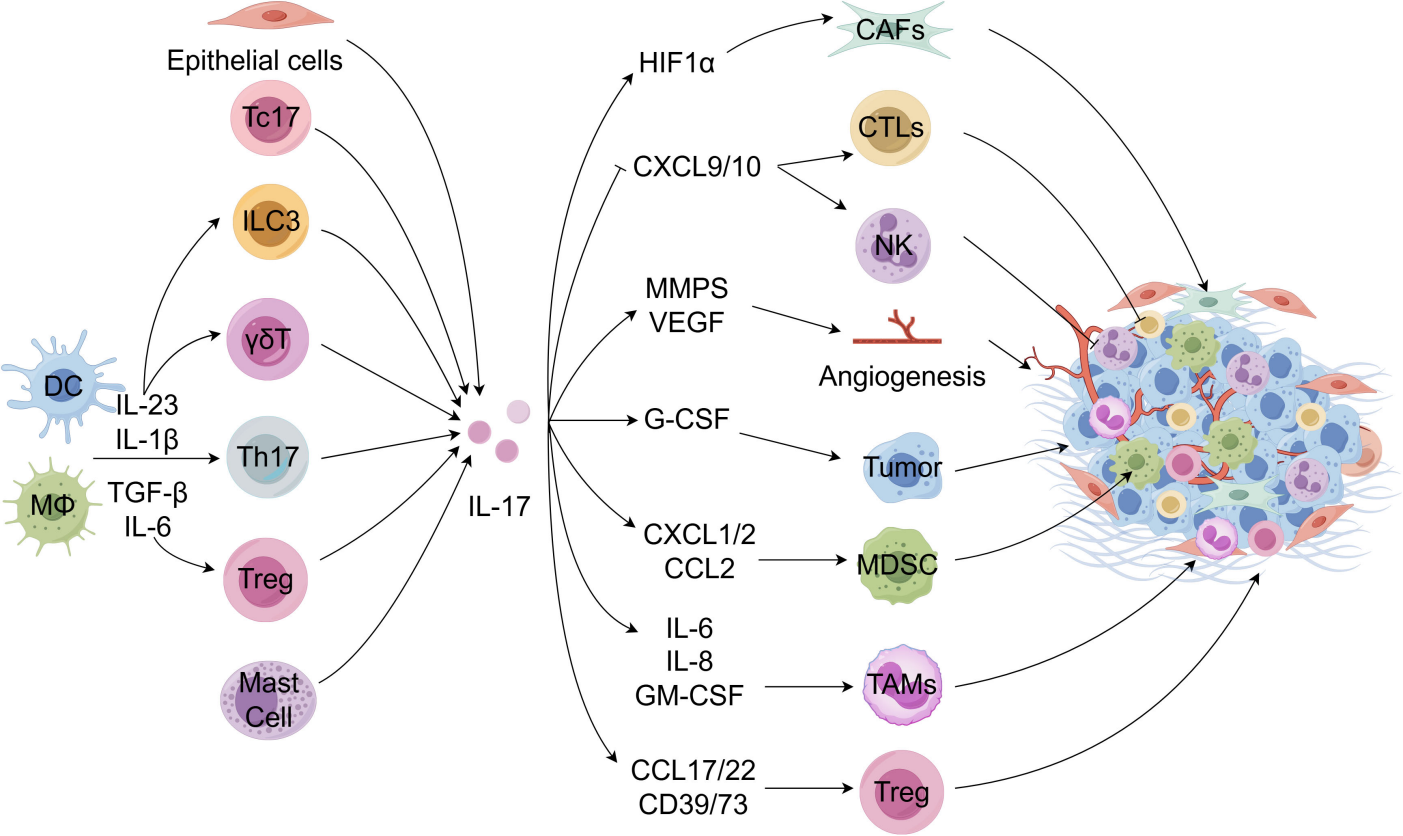
IL-17-producing cells in the TME. Tumor-infiltrating dendritic cells (DCs) and other myeloid cells are stimulated to produce various cytokines to drive the differentiation and activation of Th17 cells and other immune cells, thereby producing IL-17. Moreover, Tc17, epithelial cells, and mast cells can also produce IL-17. IL-17 acts on the tumor microenvironment in reverse. IL-17 enhances the recruitment and migration of tumor-associated macrophage (TAMs), myeloid-derived suppressor cells (MDSCs), regulatory T cells (Tregs), tumor-associated neutrophils (TANs), etc. in the TME by inducing a series of cytokines and chemokines, thereby inhibiting anti-tumor immune function. It can also increase the secretion of angiogenic factors or reduce the secretion of chemotactic factors for recruitment effect cytotoxic T lymphocyte (CTLs) and NK cells, promoting the occurrence and development of tumors. Meanwhile, IL-17 can promote anti-immune checkpoint therapy resistance by inducing collagen deposition in cancer-associated fibroblasts. FigDraw was used to generate this figure.

A diverse array of IL-17-producing cells within the TME will be elaborated upon in the subsequent discussion. **Figure 1** illustrates the presence of IL-17-producing cells within the TME.

### 2.1 Th17 cells

Characterized by their robust secretion of IL-17 and other inflammatory cytokines, Th17 cells represent a significant source of IL-17 in cancer. Orchestrated by TGF-β and IL-6, and sustained by IL-23 and IL-1β, these cells have been observed in tumors of both mice and humans (12–15). Tumor-infiltrating Th17 cells have been observed in both mice and humans (16). Th17 cells are a veritable cytokine factory, producing a range of cytokines and chemokines, including IL-17A, IL-17F, IL-21, IL-22, TNF-α, and CCL20, which modulate the behavior of fibroblasts, endothelial cells, epithelial cells, macrophages, and tumor cells, thereby sculpting the TME (17). Numerous studies have highlighted a positive correlation between Th17 cell infiltration in tumors and cancer angiogenesis (18). These cells are known to foster tumor progression by igniting angiogenesis and immunosuppressive activities. However, they can also rally immune cells into tumors, contributing to antitumor immune responses by activating effector CD8^+^ T cells (19). Therefore, Th17 cells play a multifaceted and enigmatic role in tumor immunity. Their dichotomous behavior within the TME is attributed to their plasticity, which allows for their transformation into other T cell lineages, such as Th17/Treg and Th17/Th1 cells (18). Comprehensive research into the dynamic role of Th17 cells is imperative for developing therapeutic strategies targeting these cells in cancer.

### 2.2 Tc17 cells

Several studies have documented the presence of IL-17-producing CD8^+^ T (Tc17) cells in cancer patients. Tumor-infiltrating Tc17 cells within tumors are significantly linked to poorer survival rates in gastrointestinal cancers, including gastric cancer and hepatocellular carcinomas, and unfavorable clinical outcomes in cervical cancer patients (20–22). A novel pro-tumorigenic Tc17 cell has been identified in pancreatic ductal adenocarcinoma, which accelerates tumor growth through IL-17RA-dependent modification of cancer-associated fibroblast (23). Mechanistic insights reveal that tumor-infiltrating Tc17 cells induce tumor cells to produce CXCL12, promoting the migration of MDSCs to the TME in a CXCR4-dependent manner (20). However, Tc17 cells have also demonstrated antitumor effects in a mouse model of B16 melanoma (24). The ambiguous function of Tc17 cells in cancer is partly attributed to their plasticity, as they can transition into IFN-γ-producing CTLs (25, 26). Supporting this, PD-1 has been shown to play a pivotal role in specifically suppressing Tc17 differentiation and its plasticity concerning CTL-driven tumor rejection (27).

### 2.3 Lymphoid origin

Emerging evidence underscores the pivotal role of γδ T cells in producing the cytokine IL-17 (γδ Th17) and their significant contribution to cancer progression. Studies have demonstrated that human γδ Th17 cells are instrumental in promoting tumor growth across a spectrum of human cancers, including colorectal, lung, breast, and pancreatic ductal adenocarcinoma (28–32). In human CRC, γδ Th17 cells are a predominant source of IL-17, facilitating the accumulation and expansion of PMN-MDSCs within the tumor site, thereby eliciting local immunosuppressive functions (33). Mouse models of spontaneous breast cancer metastasis have revealed that γδ Th17 cells drive TAN expansion and accumulation in a granulocyte colony-stimulating factor (G-CSF) -dependent manner, impeding effector CTL function and contributing to cancer metastasis (31). Furthermore, γδ Th17 cells have also been implicated in the recruitment of macrophages expressing high levels of IL-17 receptor, which promotes ovarian cancer cell proliferation *in vitro (* 34). The intricate crosstalk between γδ Th17 cells and the tumor-associated myeloid compartment appears to be mediated via IL-17 signaling (33). The complexity of γδ Th17 cells is continually unfolding as new insights emerge.

Tregs are associated with poor prognosis in a variety of cancers, and in addition to solid tumors (35–39), Tregs have also been considered a negative factor in the treatment of leukemia (40). However, a high “Treg signature” in human CRC indicates a better prognosis (41). It has also been found that Tregs suppress CRC development by suppressing pro-tumor inflammation (42), and ablation of Treg-related cytokines IL-10 and TGF-b leads to increased intestinal tumor burden (43). Treg cells, known for their immunosuppressive functions, can secrete IL-17, thereby modulating their interactions with other immune cells (44). IL-17 can directly signal to Tregs and promote their maturation and function, in turn, Tregs with enhanced activation and maturation markers suppress Th17 cells, resulting in decreased IL-17 production, thus forming a negative regulatory loop to control IL-17-mediated inflammation. Therefore, IL-17-mediated Treg maturation suppresses tumor-associated inflammation and reduces intestinal tumor development in early CRC (45). IL-17 can also recruit Tregs directly or enhance the expression of CCL17 and CCL22, directing Tregs to migrate and suppress anti-tumor immune functions (45, 46). Moreover, IL-17 can modulate the immunomodulatory function of Tregs by regulating their interactions with other immune cells. For instance, IL-17A can influence Treg function by affecting MDSCs and recruiting MDSCs to tumor sites via the secretion of chemokines CXCL1/2. MDSCs can, in turn, recruit Tregs through CD40 induction or cytokine secretion (47). Additional studies have demonstrated that the local inflammatory environment promotes plasticity in Treg cells, driving the differentiation of Treg cells into Th17 cells that secrete IL-17 (48). Meanwhile, Treg cytokines also reencode IL-17^+^FoxP3^+^ T cells (49), which are implicated in developing autoimmune diseases and solid cancers, including inflammatory bowel disease and esophageal, colon, and lung cancers (50).

### 2.4 Myeloid origin

Myeloid cells, most notably CD68^+^ macrophages, neutrophils, and mast cells, have also been recognized for their ability to secrete IL-17. IL-17 produced by breast cancer-associated macrophages has been reported to promote the invasiveness of breast cancer (BC) cells (51). IL-17 expression by macrophages was found to be associated with proliferative, inflammatory, atrophy-lesions in prostate cancer patients (52). TAM-derived IL-6 and IL-23 induce the production of IL-17 by the transcription factor RORγt in neutrophils. Meanwhile, as IL-17RA and IL-17RC are also expressed in TAMs, IL-17 in tumor tissue can boost the recruitment and migration of TAMs (53–55). IL17^+^ neutrophils have been observed in a variety of cancers, with high levels present in gastric cancer tissues. IL-17 induces the migration of neutrophils into gastric cancer via cancer cell-derived CXC chemokines and promotes angiogenesis through MMP9 signaling. The presence of mast cells producing IL-17 has been confirmed in various cancers, such as gastric cancer, colorectal cancer, and esophageal squamous cell carcinoma (56–58). Furthermore, IL-17-producing mast cells in esophageal squamous cell carcinoma have been suggested to function in the recruitment of effector CTLs and M1 macrophages to the tumor site, thereby serving as a favorable prognostic factor (58).

### 2.5 Epithelial and fibroblast origin

IL-17C, predominantly produced by non-immune cells such as colon epithelial cells, has been the subject of intriguing research. Studies in IL-17C-deficient mice have demonstrated that epithelial-derived IL-17 fosters neutrophilic inflammation within the TME and promotes lung tumor growth (59). IL-17 has also been shown to induce a collagen deposition program in cancer-associated fibroblasts by enhancing HIFα expression, thereby driving resistance to anti-PD-L1 (60). Moreover, IL-17 can also signal to epithelial cells and fibroblasts to produce a diverse array of chemokines and growth factors, which, depending on the tissue niche, can either fuel tumor development or aid in anti-tumor activities. For instance, IL-17 has been shown to induce the production of angiogenic factors such as VEGF, CXCL1, or CXCL8 in colorectal cancer and non-small cell lung cancer (NSCLC) patient samples (61–63). However, IL-17 has also been demonstrated to induce the secretion of CXCL9 and CXCL10, which in turn recruited effector CTL and NK cells to inhibit ovarian cancer (8).

## 3 IL-17 signaling transduction

**Figure 2:**
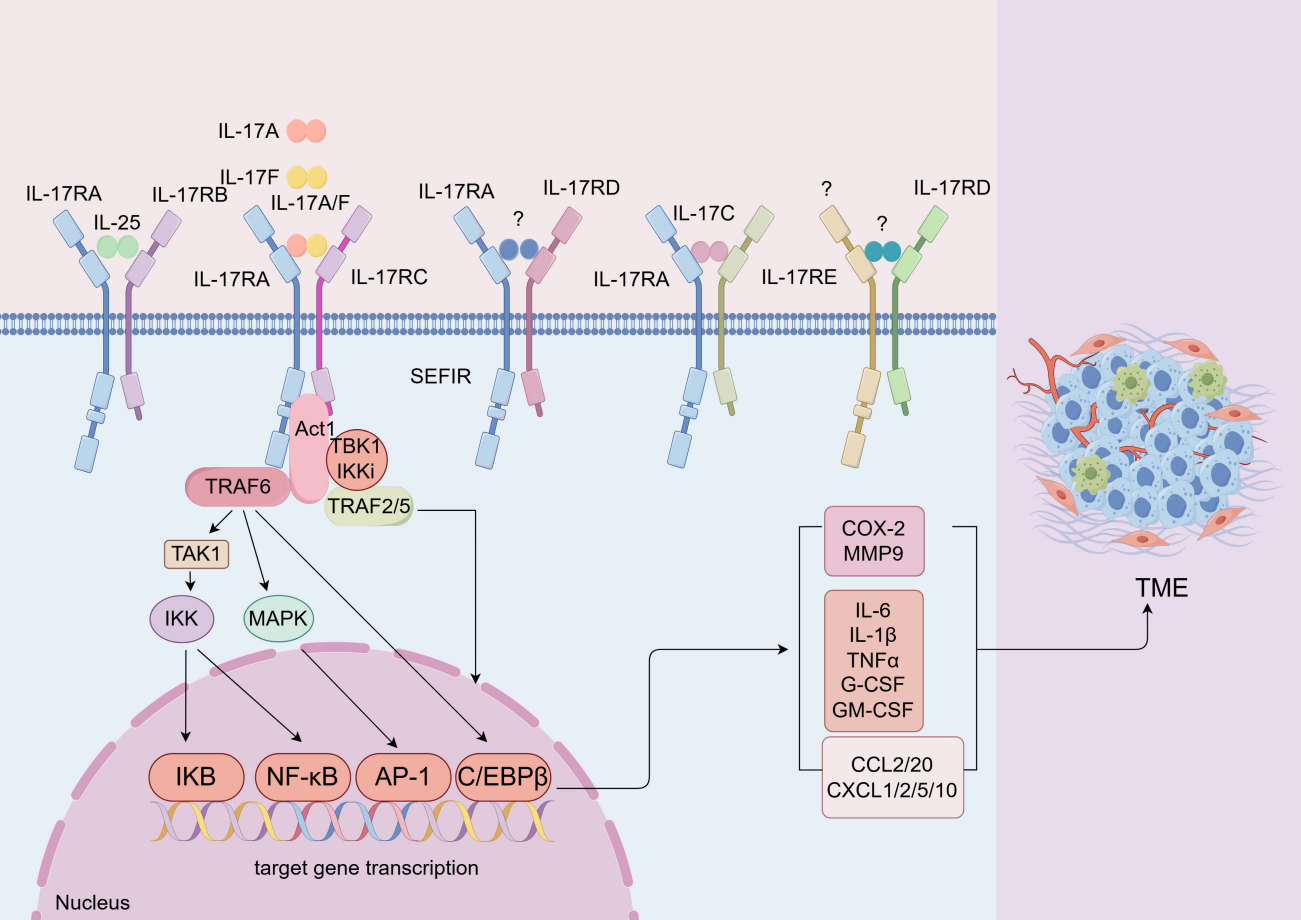
IL-17 signaling transduction. lL-17 family includes six cytokines. The lL-17 receptor family consists of five distinct receptors that share the SEFlR domain, a cytoplasmic motif (64). Different subunits combine with each other to form different heterodimer receptors. The binding of IL-17A/IL-17F/IL-17A/F to the receptor complex facilitates homotypic interactions. The combination activities mitogen-activated protein kinases (MAPK), NF-kB, and C/EBP signaling pathways through the adaptor proteins Act1 and TRAF6 (65). The activation of these pathways can also induce the secretion of inflammatory factors, chemokines, etc. FigDraw was used to generate this figure.

The IL-17 family exerts its biological effects through a network of signaling pathways as homodimers or heterodimers, with distinct but overlapping activities (**Figure 2**). IL-17A, IL-17F, and IL-17A/F heterodimers initiate qualitatively similar yet quantitatively diverse signals, with a graded potency of IL-17A> IL-17A-IL-17F> IL-17F. They engage the IL-17RA and IL-17RC complex, leading to the activation of TRAF6-dependent gene transcription (66). The initiation of IL-17 involves the recruitment of the Act1 adaptor protein to IL-17 receptors through SEFIR domain interactions, highlighting the canonical IL-17 signaling pathway’s reliance on Act1 recruitment (4). Act1 interacts with TRAF6 through its PH domains, facilitating TRAF6 ubiquitination, which in turn ubiquitinates TGF-activated kinase 1 (TAK1). This event phosphorylates IKK, culminating in IκB degradation and the activation of NF-κB and MAPK pathways: p38, ERK, and JNK (65). Notably, the Act1-TRAF6-TAK1 cascade also contributes to the activation of MAPK pathways by IL-17, essential for the activation of transcription factors such as AP-1 (4). Consequently, pro-inflammatory IL-17 signaling culminates in the transcription of target genes vital for inflammation and defense against infections, encompassing proteins like TNF, IL-6, IL-1β, IL-8, CXCL1, CXCL8, CXCL10, ICAM1, GM-CSF, and a plethora of chemokines including CXCL1, CXCL2, CXCL5, CXCL8, CXCL10, CCL2, and CCL20, as well as matrix metalloproteinases (MMPs) such as MMP1, MMP2, MMP3, MMP9, and MMP13 (67). Furthermore, TRAF2- and TRAF5-induced signaling by IL-17, controlling mRNA turnover, acts as a positive regulator of gene transcription activation (68). This pathway is contingent upon TBK1 and IκB kinase (IKKi) phosphorylation of Act1.

## 4 The role of IL-17 signaling

IL-17, recognized as an inflammatory factor, has been extensively studied for its role and mechanisms in autoimmune and inflammatory diseases. Psoriasis is the most prominent skin inflammation associated with IL-17. This cytokine drives the excessive proliferation of epidermal keratinocytes, leading to the production of additional pro-inflammatory cytokines and chemokines that sustain and amplify the inflammatory response. Currently, three IL-17 inhibitors have been approved for the treatment of psoriasis (69). Moreover, psoriatic arthritis (PsA) and ankylosing spondylitis (AS) are now understood to be activated by the dysregulated IL-23-IL-17 pathway (70, 71). IL-17 can induce the production of pro-inflammatory IL-6 and interacts with various other cytokines, such as TNF and IL-1β, to elevate inflammation levels (72). In patients with inflammatory bowel disease (IBD), both Crohn’s disease (CD) and ulcerative colitis (UC) patients exhibit high levels of IL-17 in the inflamed intestinal tissues. Research involving multiple colitis models has demonstrated that IL-17 is crucial for the maintenance of colitis (73). In addition, IL-17 and its downstream signaling pathways have been implicated in several other pathologies, including multiple sclerosis (MS), systemic lupus erythematosus (SLE), and asthma (2).

Our studies, along with others, have also suggested a correlation between inflammation and tumorigenesis (74). Evidence indicates that patients with inflammatory bowel disease are at an increased risk of developing inflammatory bowel disease-associated colorectal cancer (IBD-CRC), with inflammation serving as a key initiating factor (75). Viral hepatitis is recognized as one of the primary pathogenic factors in hepatocellular carcinoma (76). Similarly, inflammation may play a significant role in the interaction between PCa and metabolic disorders (77). Consequently, researchers have begun to explore the potential connection between IL-17 and tumorigenesis.

In addition to autoimmunity, dysregulation of IL-17 signaling is widely involved in both the early and late stages of cancer development. IL-17 may create a favorable environment for tumor development, and its expression level may be related to the recurrence rate and metastasis of tumors (46, 78, 79). IL-17A as a proinflammatory cytokine is linked to rapid malignant progression of cancer and therapy resistance (1, 64). Blocking IL-17A leads to a significant reduction of tumor growth in murine models of gastric cancer, prostate cancer, colorectal cancer, myeloma, and other type of cancer (1, 67, 80). A clinical trial is now exploring the efficacy of the anti-IL-17A antibody in multiple myeloma (81). However, IL-17F plays the opposite role from IL-17A and protects mice from colitis-associated colorectal cancer (CAC) induction (82). It has also been found that the tandem relationship between IL-17 and microbiome also affects tumor progression. The different members of the IL-17 family cytokine may exert pro-tumor or anti-tumor roles in the development of various cancers, which are associated with various TMEs.

## 5 IL-17 receptor expression profile in cancer

The IL-17R family, consisting of five receptor subunits including IL-17RA, IL-17RB, IL-17RC, IL-17RD, and IL-17RE, requires dimerization with another subunit to form functional receptors, either as homodimers or heterodimers (83). Extensive research has demonstrated the expression of the IL-17R family members in various cancers, highlighting their potential roles in tumorigenesis. Our review consolidates these findings in **Table 1**, categorizing the studies based on cancer types.

**Table 1:** IL-17 receptor expression profile in cancer.

Receptor	Tumor type	Target	Expression	Sample	Effects on tumor	Ref.
IL-17RA	GC	mRNA	High	Tumor tissue of gastric cancer patients	Correlated with tumor progression and poor prognosis of patients.	(84)
	CRC	Protein	High	Tumor tissue of CPC mice	IL-17RA promoted the occurrence and development of CRC in CPC mice.	(85)
	PCa	Protein	High	Tumor tissue of prostate cancer patients	/	(86)
	HCC	mRNA	High	Clinical HCC samples	IL-17RA promoted Alcoholic Liver Disease-induced HCC.	(87)
	OC	Protein	High	Tumor tissue of tongue tumor-bearing mice infected with Candida albicans	IL-17A/IL-17RA induced CCL2 and attracts macrophages into the tumor environment.	(55)
IL-17RB	GC	mRNA and Protein	High	Tumor tissue of gastric cancer patients	The over-expression of IL-17RB is associated with poor prognosis and contributes to gastric cancer cells acquiring stemness.	(88)
	LCM	mRNA	High	Tumor tissue of lung adenocarcinoma patients	IL-17RB cancer cell promoted invasion, migration, and metastasis.	(89)
	PCa	Protein	High	Tumor tissue of pancreatic cancer patients	High expression led to tumorigenesis, metastasis, and poor prognosis.	(90, 91)
IL-17RC	GC	Protein	High	rhIL-17A-treated AGS cells and SNU 601 cells、Human gastric cancer tissue	IL-17A/IL-17RC regulated GC development by modulation of the NF-κB/NOX1.	(80)
	NSCLC	Protein	High	Tumor tissue of NSCLC patients	/	(92)
	PCa	Protein	High	RWPE-1, pRNS-1-1, MLC-SV40, LNCaPc cell lines; Tumor tissue of prostate tissue patients	/	(93, 94)
IL-17RD	CRC	Protein	High	Tumor tissue of CRC patients	Promoted colitis-associated tumorigenesis and is negatively correlated with survival rates in mice and colorectal cancer patients.	(95)
	PCa	Protein	Low	LNCaP, PC3, LN3, PC3M, DU145 cell lines	Enhanced the tumorigenic response.	(96)
	BC	mRNA	Low	MDA-MB-23, MCF-7 cell line	/	(97)
	Ulcerative colitis tumors	Protein	High	Tissue of ulcerative colitis patients with neoplasia	Promoted carcinogenesis.	(98)
IL-17RE	HCC	Protein	High	Tumor tissue of HCC patients	The high expression of IL-17 and IL-17RE in tumors is significantly associated with reduced survival and increased recurrence rates in HCC patients.	(99)
	BC	mRNA	High	MCF7, MDA-MB361, T47D, and ZR75 cell lines, MCF10A, MDA-MB468, MB435-S, MB231, MB175-7, SKBR3, HS578T, HBL100, and HCC1937 cell lines	IL-17RE mediated cell death signaling of IL-17E.	(100)

### 5.1 IL-17RA expression in cancer

IL-17RA, initially identified as the receptor for IL-17A, also binds to IL-17F and the IL-17A/F heterodimer. It is currently the most extensively studied member of the IL-17R family. Overexpression of IL-17RA in gastric cancer (GC) tissue compared to normal adjacent tissues suggests its significant role in the progression, metastasis, and prognosis of gastric cancer may play an important role (84). Contrarily, in colorectal cancer (CRC) samples, IL-17RA expression was significantly reduced, correlating with the expression of A_20_, a key negative regulator of NF-κB and JNK-c-Jun pathways (85). However, our previous studies indicated that IL-17RA is highly expressed in mouse tumor tissues, promoting CRC occurrence and development. Deletion of IL-17RA enhances the recruitment of CD8 cytotoxic T lymphocytes (CTLs) to suppress tumors (101). This discrepancy in the expression profiles between humans and mice underscores the complexity of IL-17RA’s role. Additionally, IL-17RA within transformed colon epithelial cells has been shown to activate ERK, p38 MAPK, and NF-κB signal transduction, promoting the proliferation of oncogenic intestinal cells that have lost APC tumor suppressor factors (102). Compared with normal cells, IL-17RA phosphorylation in proliferative human prostate cancer cells decreased, while IL-17RA levels increased (86). Furthermore, IL-17RA is upregulated in the liver tissue of alcohol-induced HCC mice and ALD-related liver fibrosis and HCC patients. *IL-17RA^-/-^* mice are protected from EtOH+HFD-induced HCC (87). Studies have revealed that the IL-17A/IL-17RA signaling pathway is activated in oral cancer cells infected with Candida albicans, which induces CCL2 and attracts macrophages into the tumor environment (55). Fibroblasts express IL-17RA in PDAC and promote Tc17-driven tumor growth (23). IL-17A/IL-17RA induces increased expression of FABP4 through p-STAT3 in the presence of palmitic acid (PA), promoting OvCa cell proliferation (103).

### 5.2 IL-17RB expression in cancer

IL-17RB expression is significantly elevated in human gastric cancer tissue and is associated with poor prognosis, contributing to gastric cancer cells acquiring stemness (88). Overexpression of IL-17RB in lung cancer tissue significantly increases cancer cell invasion, migration, and metastasis (89). Studies on human prostate cancer tumor specimens revealed overexpression of IL-17RB and phosphorylated IL-17RB, playing a significant role in tumorigenesis and metastasis of pancreatic tumors (90, 91).

### 5.3 IL-17RC expression in cancer

IL-17RC is upregulated in helicobacter pylori-induced gastric cancer tissues and human cancer tissues, participating in the IL-17A/IL-17RC axis and modulating the development of GC through the NF-κB/NOX1 signaling pathway (80). The immune reactivity of IL-17A, IL-17F, IL-17RA, and IL-17RC in the lung sections from NSCLC was significantly higher than that in normal control (92). Evidence suggests differential expression of IL-17RC protein isoforms in prostate cells and cancer tissues (93, 94). In another study, immunohistochemistry was performed on prostate tumor tissues from 40 patients revealing that IL-17RC levels were higher than the IL-17RA levels across all histological grades of the Gleason score (104).

### 5.4 IL-17RD expression in cancer

Research has confirmed that IL-17RD acts as a tumor suppressor factor in mice, primarily by limiting the duration and severity of inflammation (105). IL-17RD/Sef, as a regulatory factor for T cell subsets, promotes colitis-associated tumorigenesis and is negatively correlated with survival rates in mice and colorectal cancer patients (95). IL-17RD is downregulated in advanced prostate cancer, potentially enhancing the tumorigenic response to fibroblast growth factors (FGF) (96). In human breast, prostate, thyroid, and ovarian tissues, IL-17RD is highly expressed in surface normal epithelial cells but significantly downregulated in most tumors originating from these epithelia (106). The expression of IL-17RD in breast tumor cells is significantly downregulated compared to normal breast cells, which is related to EMT (97). Interestingly, research by Joel Pekow et al.’s research suggests that in ulcerative colitis tumors, the expression of miR-193a-3p, a key tumor suppressor factor, is downregulated, and its deficiency promotes carcinogenesis by upregulating IL17-RD (98).

### 5.5 IL-17RE expression in cancer

Among the IL-17 receptor family, IL-17RE expression in cancer is less understood. Postoperative studies of patients with HCC revealed a significant correlation between high expression of intra-tumoral IL-17A, and IL-17RE with decreased survival and increased HCC patients’ recurrence rates (99). The expression of IL-17RE in tumors of patients with poor prognosis is much higher than in nonmalignant breast tissue, and this differential expression mediates the apoptotic activity of IL-17E (100).

## 6 IL-17 signaling in cancers

We delve into the intricate mechanisms by which IL-17 influences the genesis and progression of inflammation-associated cancers, compiling the findings in **Table 2**.

**Table 2:** IL-17 signaling in cancers.

Tumor type	IL-17 type	Cell lines/animal type	Effect	Mechanism	Refs.
CRC	IL-17A	*Il17ra* ^−/−^ *Cdx2^-^Cre* ^+^/*Apc* ^F/+^ mice	Pro-tumor	Promoted the proliferation of transformed colonic epithelial cells by activating ERK, p38 MAPK and NF-κB signaling.	(102)
		*Il17ra* ^+/−^ *Il17ra* ^−/−^ *Cdx2* ^-^ *Cre*/*Apc* ^F/+^ mice; *Cdx2-Cre^ERT2+^/Apc^F/F^* mice	Pro-tumor	Inhibited their production of CXCL9/10 chemokines to decrease the infiltration of CD8^+^ CTLs and Tregs to CRC.	(101)
		Clinical CRC patients	Pro-tumor	Promoted the infiltration and development of MDSCs to inhibit the activity of CTLs.	(107)
		Clinical CRC patients; CRC cell lines: SW480, HCT116 and HT-29	Pro-tumor	Stimulated endothelial cell migration and the production of proangiogenic factors.	(108)
		M38 xenografted *Il17^-/-^* C57/BL6 mice	Anti-tumor	Decreased IFN-γ^+^ CD4^+^ and IFN-γ^+^ CD8^+^ T cells in tumor tissue.	(109)
		Clinical CRC patients; A model mimicking human colon cancer; CRC cell lines: SW620 and HT29	Anti-tumor	Induce mitochondrial dysfunction and pyroptosis through ROS/NLRP3/caspase-4/GSDMD pathway and promote intracellular ROS accumulation. Promote the secretion of IL-1b, IL-18 and other inflammatory factors and immune antigens, and recruit CD8^+^ T cells.	(110)
	IL-17F	CAC mouse model	Anti-tumor	Maintained the barrier integrity in epithelial cells.	(111)
BC	IL-17A	Human TNBC cell lines: MDA-MB-231/468 cell; *In situ* model with 4T1 in *Il17^-/-^* immunodeficient NU-Foxn1nu mice	Pro-tumor	Directly promote the migration and angiogenic activity of breast cancer tumor cells and enhance anoikis resistance.	(112)
		Spontaneous metastasis model: K14cre/Cdh1^F/F^/Trp53^F/F^ mice	Pro-tumor	Stimulated G-CSF to increase neutrophil polarization.	(31)
		Clinical BC samples; 4T1 tumor-bearing mice	Pro-tumor	Activated signaling pathways such as STAT3, NF-κB, and ERK1/2 in tumor cells.	(113)
		*In situ* tumor model with Cl66, Cl66-Dox, and Cl66-Pac cells	Pro-tumor	Through the CXCL1/5-CXCR2 axis recruitment MDSCs.	(114)
		Clinical BC samples; TNBC cell lines: MDA-MB-231 and HCC-38	Pro-tumor	Induces neutrophil infiltration.	(115)
	IL-17B	MCF7 or MDA-MB-468 cells xenografted nude mice; BC cell line: MDA-MB-468 and MCF7	Pro-tumor	Activated the ERK and NF-κB pathway in the tumor and enhanced the expression of Bcl-2 in the tumor.	(116)
	IL-17E IL-17A	BC cell line: MCF7, MCF10A; IJG-1731	Pro-tumor	Induce c-RAF phosphorylation, ERK1/2 and p70 S6 kinase are involved in the proliferation and survival of tumor cells. Exacerbated breast cell resistance to docetaxe. Induction of cyclin E (LMW-E) production.	(117)
	IL-17A	BC cell line: MCF-7, MDA-MB468; T47D	Anti-tumor	Activated caspase-mediated apoptosis in breast tumor cells.	(118)
Lung cancer	IL-17A	IL-17A knockdown and overexpression in A549 and SPC-A-1 cell; Anti-IL17A treatment in LLC tumor bearing	Pro-tumor	Inhibited the apoptosis through the ROS/Nrf2/p62 pathway leading to increased PD-L1 expression in NSCLC cells.	(119)
		Clinical NSCLC samples; the human lung cancer cell line A549; Sq-19IL-17 or A549IL-17 xenografted SCID mouse	Pro-tumor	Activated STAT3, NF-κB, JAK/STAT pathway, and CXCR-2 in tumor.	(62, 120)
		Clinical NSCLC samples; A549 and H460 cells; H460 cells transfected with IL-17-expressing xenografted nude mice	Pro-tumor	Mediated the migration, invasion and dryness of NSCLC through STAT3/NF-κB/Notch1 signaling.	(121)
		3LL or B16-F10 xenograft IL-17A^KO/WT^ mice; NSCLC cell lines: A549, LLC	Pro-tumor	Up-regulated the expression of IL-8, MMP2, MMP9, p-STAT3, and ZEB1 in tumor.	(60, 122–125)
		Serum of clinical NSCLC samples; *Il17a* and *Kras^G12D^* in mice	Pro-tumor	Up-regulated the expression of G-CSF through the NF-κB pathway in tumor, which recruits MDSCs.	(126)
		Clinical NSCLC samples; NSCLC cell lines: A549, L3, L4	Pro-tumor	Promoted the secretion of CCL20 by cancerous cells.	(127)
		Clinical NSCLC samples; Human lung adenocarcinoma cell lines	Pro-tumor	Promoted angiogenesis by stimulating VEGF production of cancer cells via the STAT3/GIV signaling pathway in non-small-cell lung cancer.	(61)
	IL-17D	IL-17D–overexpressing LLC1 cells xenograft C57BL/6 mice	Pro-tumor	Induced tumor-associated macrophage infiltration via the p38 MAPK signaling pathway in tumor.	(53)
	IL17A	*Il17* ^−/−^ in MPE mice	Anti-tumor	Activated the STAT1 pathway in cancer.	(128)
		LLC xenograft *Il17^-/-^* nude mice	Anti-tumor	Maintained the killing effect on the T cells and combination with PI3K pathway inhibitors and Toll-like receptor agonists.	(129)
Liver cancer	IL-17A	Ectopic allograft of IL-17-expressing LPCs and DEN+CCl_4_ murine model; Clinical HCC samples	Pro-tumor	Stimulated LPC promotes their transformation into CSCs through miR-122 downregulation.	(130)
		C57BL/6 mice with a high-fat diet	Pro-tumor	Increased lipid uptake and impairing cholesterol and fatty acid synthesis in hepatocytes.	(131)
		*Il17ra ^KO/WT^* in alcohol-induced HCC models; Clinical HCC samples	Pro-tumor	Activated the caspase 2-S1P-SREBP1/2 pathway to up-regulate the expression of DHCR7 and FASN in cancer.	(87)
		Clinical HCC samples; MHCC-97L	Pro-tumor	Increased the expression of MMP2 and MMP9 through the NF-κB pathway and AKT2/STAT3 pathway in tumors.	(132, 133)
		Clinical HCC samples; IL-17-treated normal liver cells or liver cancer cells or co-culture; Anti-Gr1 antibody to delete neutrophils in hepatoma-bearing mice	Pro-tumor	IL-17 may promote the migration of neutrophils into HCC and then neutrophils can stimulate the proangiogenic activity of tumor cells.	(134)
		Clinical HCC samples; SMMC7721-IL-17 or SMMC7721-mock tumor cells xenograft nude mice	Pro-tumor	Activated the JAK2/STAT3 signaling pathway and the production of IL-6. Up-regulation of IL-8, MMP2, and VEGF.	(132)
		Clinical HCC samples; HCC cell line: Human HepG2 and Huh7	Pro-tumor	Inhibited the anti-tumor effect of IFN-γ and up-regulate the expression of negative feedback regulator PIAS1 by activating the JAK/STAT1 pathway.	(135)
		Clinical HCC samples; Human HCC cell line: SMMC-7721 and HepG2	Pro-tumor	Decreased the expression of cyclin D1.	(136)
	IL-17A	HepG2 cells after transduced with lentiviruses encoding Beclin1; Human HCC cells line HepG2	Anti-tumor	IL-17A inhibited the autophagic activity of HCC cells by inhibiting the degradation of Bcl2.	(137)
	IL-17F	Transfected SMMC-7721 human hepatocarcinoma cells with RV-IL-17F; SMMC-7721/RV-IL-17F xenograft nude mice	Anti-tumor	Inhibited the vascular endothelial cells and decreased the expression of angiogenesis factors.	(138)
PCa	IL-17A	*Pten/Trp53* mouse models; Human PCa cells	Pro-tumor	Promoted epithelial to mesenchymal transition by MMP7.	(139, 140)
		Human prostate cancer cell line	Pro-tumor	Up-regulated the expression of PD-L1 by the NF-κB and ERK1/2 signaling.	(141)
		Clinical prostate samples; Benign prostatic hyperplasia cells (BPH) and various PC cell lines	Pro-tumor	Increased the expression of CTSK through the IL-17/CTSK/EMT axis.	(142)
		HUVECs and human PC cell lines	Pro-tumor	Increased the expression of VCAM-1 in vascular endothelial cells through CD44-VCAM-1 interaction.	(143)
		*E.G7-OVA or Il17ra^−/−^* tumor bearing mice	Pro-tumor	Induced the infiltration of MDSCs.	(144–146)
		*Il17rc^KO/WT^* mice	Pro-tumor	Activated the COX-2-VEGF pathway.	(147)
	IL-17F	Prostate cancer cell line: PC-3 and DU145	Pro-tumor	Actived the PI3K/AKT signaling pathway.	(148)
OC	IL-17A	*In situ* model with ID8 cells in *Il17^-/-^* mice; Clinical OC samples; A2780, OVCAR3 and SKOV3 cells	Pro-tumor	Promote the growth and metastasis of OvCa by increasing the expression of FABP4 and p-STAT3.	(103)
		Ovarian CD133 CSLCs	Pro-tumor	Promoted the self-renewal of CD133 CSLC through NF-κB and p38 MAPK signaling pathway.	(149)
		Clinical OC samples; the OVSAHO human serous carcinoma cell line and the MCAS mucinous carcinoma cell line	Pro-tumor	Induced the expression of PD-L1 in tumors.	(150)
		Anti-IL17A treatment in the syngeneic murine model; Clinical OC samples	Pro-tumor	Made TNF receptor 1 (TNFR1) proinflammatory cytokines to promote tumor development.	(151)
		CAOV-3 and OVCAR-3 cells; SKOV3 cells xenograft nude mice; Clinical OC samples of serum	Pro-tumor	Up-regulated the expression of metastasis-associated 1MTA1 in tumors.	(152)
		Clinical OC samples; IL‐17A treated murine omenta cell in mice	Pro-tumor	Increased the recruitment of MDSCs in tumors.	(153)
		Clinical OC samples	Pro-tumor	Promoted the production of blood vessels and inhibited CXCL9 and CXCL10 in ovarian cancer cells.	(8)
	IL-17A	Clinical OC samples	Anti-tumor	Recruited CD4^+^ T and CD8^+^ T cells CXCL9 and CXCL10 can induce effector cells.	(7)
GA	IL-17A	Clinical GC samples	Pro-tumor	Promote tumor progression through IL17, IL22 and IL26 signaling or Tc17 (IL-17CD8 T cells) differentiation into exhausted T cells.	(154)
		Human gastric cancer cell line MGC-803; IL-17-treated quiescent gastric CSCs xenograft in nude mouse	Pro-tumor	Activates STAT3 signaling to promote tumor and VEGF-A expression and neovascularization.	(155, 156)
		Clinical GC samples	Pro-tumor	Recruit neutrophils and promote invasive margin angiogenesis.	(67, 157)
		Human gastric cancer cell line MGC-803; IL-17 treated quiescent gastric CSCs xenograft in nude mouse	Pro-tumor	Activating JAK2/STAT3 signaling pathway promotes EMT, migration, and invasion of gastric cancer cells, and promotes the transformation of quiescent gastric CSCs into invasive gastric CSCs.	(155)
		*Il17A ^KO/WT^* mice	Pro-tumor	Activates the NF-κB/NADPH oxidase 1 (NOX1) signaling pathway and promotes the cell cycle transition from G1 to S phase.	(80)
PDAC	IL-17A	Anti-IL17A treatment in the xenograft/orthotopic PDAC mice with KPC	Pro-tumor	Recruiting neutrophils, triggering neutrophil extracellular traps (NETs), and eliminating CD8 T cells from the tumor.	(158)
		Anti-IL17A treatment in the Pdx1-Cre (KPC) mice; Clinical pancreatic tumor samples	Pro-tumor	Regulate cluster cell development and stem cell characteristics of pancreatic cancer cells by increasing the expression of DCLK1, POU2F3, ALDH1A1, and IL17RC.	(159)
		Clinical Pancreatic Tumor samples; Genetically Engineered Mice *:Il17ra* ^-/-^ *Il17rc* ^-/-^ *Il17rafl/fl*; Anti-IL-17 treatment in the orthotopic pancreatic tumor model; Murine PDAC KPC (KPC*^Il17ra+/+^*) (LSL-*Kras^G12D/+^*; LSL-*Trp53^R172H/+^*;*Pdx1*-Cre) cells	Pro-tumor	Increases DUOX2 signaling in tumor cells.	(9)
		Human and murine PDAC cells; Clinical pancreatic tumor samples; PDAC cell xenograft Balb/c; Recombinant AAV orthotopic *Kras^G12D^* mice	Pro-tumor	Promote PanIN/PDAC by up-regulating Notch activity through the classical NF-κB pathway *in vitro*.	(160)
		Anti-IL-17 treatment in the Pdx1-Cre; Kras^G12D/WT^ mice; MiaPaCa2, Panc1, and THP-1 cells	Pro-tumor	Stimulate the pancreatitis mediator to regenerate islet-derived 3-β, leading to STAT3 activation, thereby promoting PDAC development.	(161)
	IL-17B	Cell lines:MiaPaCa-2、Panc-1 and HPAF-II; Clinical pancreatic tumor samples; IL-17RB overexpressing PSCs xenograft in NOD-scid IL-2Rgamma^null^ mice	Pro-tumor	Activate ERK1/2, enhance the production of a series of chemokines, such as C-C motif chemokine ligands, and ultimately promote the recruitment of neutrophils, lymphocytes, and endothelial cells.	(162)
		Human pancreatic cancer cell lines; Clinical pancreatic tumor samples; GFP-LUC-tagged CFPAC-1 cells orthotopic in NOD/SCIDγ mice	Pro-tumor	Enhance PDAC tumor cell metabolism and proliferation.	(91)
ESCC	IL-17A	The human esophagus adenocarcinoma cell line OE19	Pro-tumor	Promote the migration and invasion of EAC cells through ROS/NF-κB/MMP-2/9 signaling pathway activation.	(163)
		Clinical ESCC patients	Anti-tumor	Recruitment of effector CTLS, M1 macrophages, NK cells, T cells, and dendritic cells.	(164, 165)
		Clinical ESCC patients	Anti-tumor	Enhance the migration and killing ability of B cells.	(165)
		Clinical ESCC patients; ESCC cell lines: EC109 and KYSE30	Anti-tumor	Stimulated ESCC tumor cells to release more CXC chemokines CXCL2 and CXCL3 and enhanced the killing ability of neutrophils.	(166)
Melanoma	IL-17A	Anti-IL-17 treatment in the xenograft IL-17^−/−^ mice with B16-F10 tumor cells	Pro-tumor	Inducing IL-6 expression and activating Stat3 signaling.	(167)
		Xenograft B16F10 in Foxp3 DTR mice	Pro-tumor	Promoting the terminal exhaustion of CD8 T cells.	(168)
		Two human melanoma cell lines (A375 and A2058) and a mouse melanoma cell line (B16F10); Clinical melanoma patients	Pro-tumor	Induces EPHA5 via TRAF2 to recruit PIAS2 and ELAVL1 to promote melanoma development.	(68)
		Clinical melanoma patients; Human melanoma cell lines with the BRAFV600 mutation and murine cell lines; Anti-IL-17 treatment in the xenograft mice with mouse melanoma cells	Anti-tumor	IL-17 can promote the activation of T cells and neutrophils, contributing to the clinical benefit of immune checkpoint therapy.	(169)
HNC	IL-17A	SAS cells; Clinical HNC patients	Pro-tumor	Promote the proliferation of human oral squamous carcinoma cells by producing IL-6 and VEGF-A.	(170)
NB	IL-17A	Neuroblastoma cell lines SH-SY5Y (CRL-2266) and SK-N-BE2 (CRL-2271); Clinical NB patients	Pro-tumor	Producing γδT cells may produce some specific factors, mainly IL-17A, which may play a pro-tumor role.	(171)

### 6.1 Pro-tumor functions of IL-17 in colorectal cancer

Tumor-infiltrating Th17 cells have been identified in CRC (172). In individuals with stage I/II CRC, a pronounced “Th17 signature” is associated with significantly reduced disease-free survival following the resection of primary tumors (173). Elevated levels of IL-17 cytokines have been detected in both the sera and tumor tissues of CRC patients (174). In the AOM-DSS-induced CAC mouse model, IL-17A plays a crucial role in the progression of colorectal tumors (175). Notably, the neutralization of IL-17A through antibody-mediated methods has been shown to inhibit tumor development in this CAC model (176). Additional studies indicate that the genetic deletion of either IL-17A or IL-17F can also impede tumor development in APC^Min^ tumor mice (177, 178).

However, the APC^Min^ mouse model develops microadenomas in the small intestine rather than the distal colon and does not represent the most accurate CRC model (179). A more accurate model of CRC is provided by the so-called CPC-APC mouse where one allele of the APC tumor suppressor gene is deleted in the colon and loss of the second allele through loss-of heterozygosity (LOH) results in the development of large colonic adenomas that can progress to invasive carcinomas (180). Employing this model, our studies have shown that the ablation of IL-17RA inhibited colon tumor development and progression in the CPC-APC mouse model (102). These results indicate that IL-17 signaling has a protumor role in colon cancer, and that neutralization of IL-17A could potentially inhibit tumor progression and promote tumor sensitivity to chemotherapy (102).

Many studies have shown different mechanisms of IL-17 signaling to exert pro-tumor action. Firstly, L-17RA signaling promotes the proliferation of transformed colonic epithelial cells by activating ERK, p38 MAPK, and NF-κB signaling stimulation and promotes early CRC tumor development (102). Secondly, IL-17 signals to colorectal tumor cells and inhibits their production of CXCL9/10 chemokines (101). Thus, IL-17 inhibits the infiltration of CD8^+^ CTLs and Tregs to CRC, promoting CRC development (101). Additionally, IL-17 directly stimulates Tregs and promotes their maturation and function. However, this signaling pathway constitutes a negative feedback loop that controls cancer-promoting inflammation and prevents cancer development in CRC (45). Finally, IL-17 has also been shown to promote the infiltration and development of MDSCs that can inhibit the activity of CTLs and promote tumor development (107). IL-23 produced by tumor-associated myeloid cells also promotes IL-17 response and tumor growth (173, 181). Meanwhile, IL17A also proved to stimulate endothelial cell migration and the production of proangiogenic factors (64, 108).

As a crucial regulator of the intestinal microenvironment, the intestinal microbiota exerts significant regulatory effects on IL-17 secretion (182). Segmented filamentous bacteria (SFB) have been shown to induce the release of serum amyloid A (SAA) protein and enhance IL-17 production (183). Recent studies indicate that SFB flagellin can stimulate Th17 cells to promote IL-17 secretion (184). Increasing evidence suggests that the crosstalk between gut microbiota and IL-17 contributes to the development of CRC. IL-17 induces the defect of the tumor surface barrier, which makes it easier for intestinal microorganisms to enter the tumor, further promotes the secretion of inflammatory factors, and promotes the occurrence and development of tumor-related inflammation (74). Research has demonstrated that cathepsin K (CTSK), produced following dysbiosis of the gut flora, stimulates M2 tumor-associated macrophages (TAMs) to secrete IL-17, subsequently activating the NF-kB pathway and facilitating CRC invasion and metastasis (185). Furthermore, indole-3-lactic acid (ILA), a metabolite derived from L. reutii upon gut enrichment, targets ROR**γ**t to inhibit Th17 cell differentiation and downregulate the IL-17 signaling pathway, thereby suppressing CRC development (186). Additionally, blockade of IL-17 signaling also prevented enterotoxigenic *Bacteroides fragilis* (ETBF)-induced colitis and tumorigenesis in the mouse model of CAC induced by ETBF infection of APC^Min^ mice (102). Furthermore, in intestinal epithelial cells (IECs), microbiota in intestinal tumors up-regulates IL-17C through Toll-like receptor (TLR) -MyD88-dependent signaling, and microbiota-driven IL-17C induces Bcl-2 and Bcl-xL expression in IECs in an autocrine manner. Then promoting cell survival and tumorigenesis in chemically induced and spontaneous intestinal tumor models (187). Therefore, investigating the interplay between IL-17 and microorganisms may provide valuable insights for enhancing chemopreventive strategies against CRC.

### 6.2 Anti-tumor functions of IL-17 in colorectal cancer

**Figure 3:**
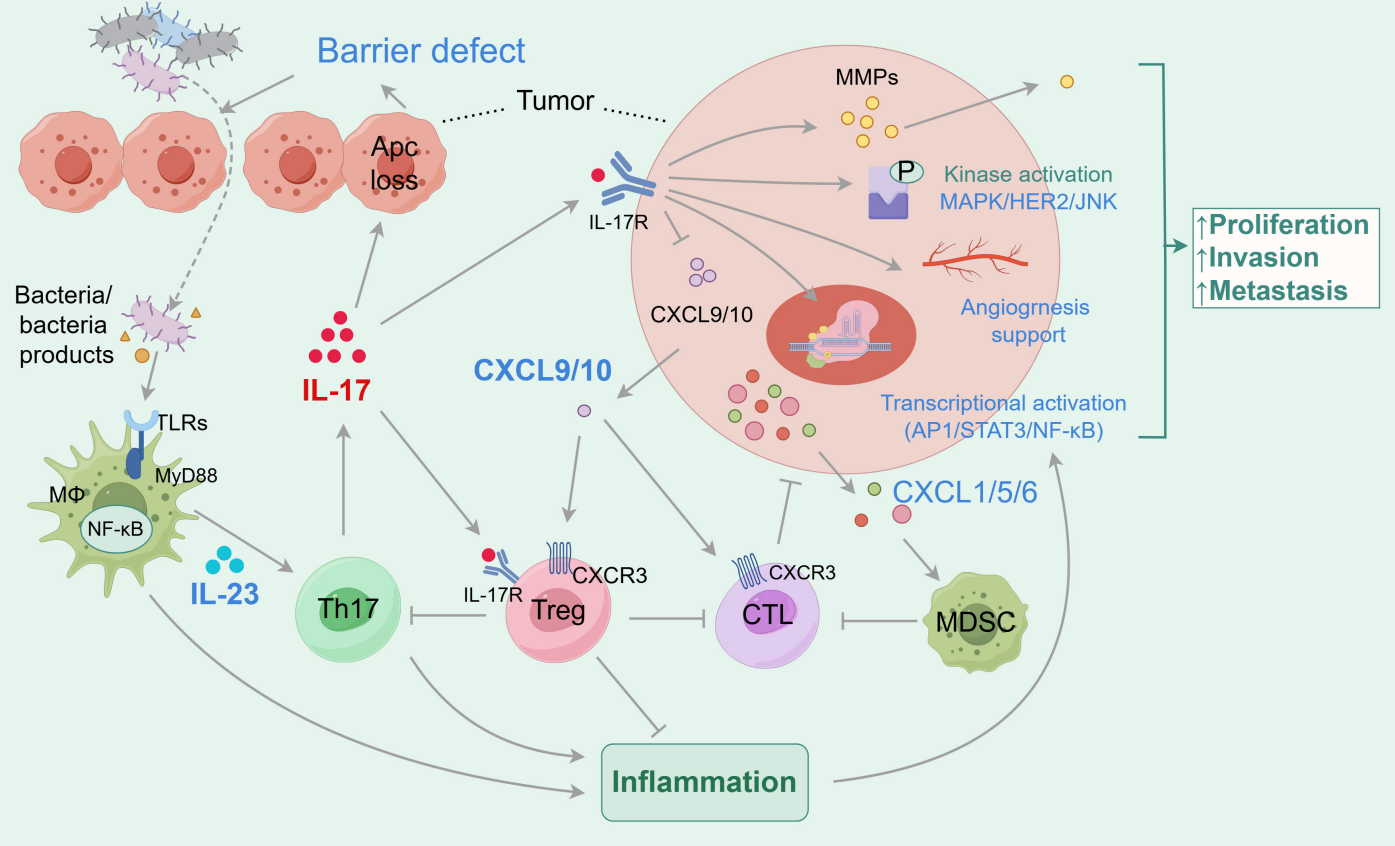
The relationship between IL-17, gut microbiota, inflammation and colorectal cancer. In the tumor microenvironment, the secretion of IL-17 is related to the gut microbiota. The gut microbiota and its secretions activate TLR receptors on macrophages, which secrete IL-23 to stimulate Th17 cells to secrete pro-inflammatory cytokine IL-17. IL-17 can directly bind to receptors on tumor cells. On the one hand, IL-17 induces defects in the tumor surface barrier, making it easier for gut microbiota to enter the tumor, promoting the secretion of inflammatory factors, and facilitating the development of tumor-associated inflammation, achieving a pro-tumor effect; On the other hand, it promotes tumor cell proliferation, invasion, and migration by activating molecular signaling, tissue remodeling, and angiogenesis. The indirect tumor-promoting effect of IL-17 is related to the recruitment of immune cells and immunosuppressive cells. IL-17 promotes tumor development by inhibiting the secretion of CXCL9/10, which recruits CD8^+^CTLs and Tregs to infiltrate CRC. IL-17 has also been shown to promote the infiltration and development of MDSCs, inhibit CTL activity, and promote tumor development. In addition, IL-17 directly acts on Tregs, thereby inhibiting Th17 and forming a negative feedback loop.

Despite the potential oncogenic role of IL-17, studies also suggested the anti-tumor function of IL-17 in colon cancer (188). Ablation of IL-17 resulted in accelerated tumor growth and more metastatic foci of tumors in subcutaneous inoculation of MC38 colonic-tumor mouse model, in which IFN-γ^+^ CD4^+^ and IFN-γ^+^ CD8^+^ T cells were decreased in tumor tissue (109). This suggests the opposite observation compared to the genetic deletion tumor model and may be explained by the subcutaneous tumor model not portraying the most accurate tumor immune microenvironment. At the same time, it was found that IL-17A could induce mitochondrial dysfunction and pyroptosis through ROS/NLRP3/caspase-4/GSDMD pathway and promote intracellular ROS accumulation in CRC patients. In addition, IL-17A can promote the secretion of IL-1β, IL-18, and other inflammatory factors and immune antigens, recruit CD8 ^+^ T cells to infiltrate the tumor and inhibit the progression of CRC (110). Furthermore, IL-17F has been shown to play a protective role in mouse CAC induction, which is associated with the important role of IL-17F in maintaining the barrier integrity in epithelial cells in the murine colitis model (111). The diverse functions of IL-17A and F cytokines in CAC may be explained by their distinct roles in autoimmune and chemically induced inflammation, which is a pivotal step in CAC induction (189). **Figure 3** reveals this relationship between IL-17, gut microbiota, inflammation, and colorectal cancer.

### 6.3 Breast cancer

It has been indicated that levels of IL-17, IL-6, and G-CSF cytokines are significantly elevated in patients with breast cancer (190). It has also been shown that IL-17A can directly promote the migration and angiogenic activity of breast cancer tumor cells and enhance anoikis resistance (112). IL-17 signaling can stimulate systemic G-CSF, increasing neutrophil polarization, and promoting breast cancer metastasis (31). IL-17A promotes tumorigenesis, proliferation, angiogenesis, and chemotherapy resistance by activating signaling pathways such as phosphorylated STAT3, NF-κB, and ERK1/2 (113). Furthermore, IL-17 also enhances the inhibitory effect of MDSCs through the CXCL1/5-CXCR2 axis (114). This results in promoting angiogenesis, tumor proliferation, and chemoresistance in breast cancer. IL-17 not only interacts with MDSC but also induces neutrophil infiltration through CXCL1 to promote the progression of triple-negative breast cancer (TNBC) (115). Similar to IL-17A, IL-17B has been shown to accelerate breast cancer development by activating the ERK and NF-κB pathway and by enhancing the expression of anti-apoptotic Bcl-2 family members (116). Some studies have found that IL-17E can induce c-RAF phosphorylation, ERK1/2 and p70 S6 kinase are involved in the proliferation and survival of tumor cells, aggravation of docetaxel resistance of breast cells, and induction of cyclin E (LMW-E) production, whose high level is closely related to the poor survival rate of breast cancer patients. In this study, IL-17A was found to have the same effect (117).

Whereas other studies observed the caspase-dependent pro-apoptotic effect of IL-17E on cultured tumor cells and xenografts (100). Consistently, recombinant IL-17E leads to an anti-tumor effect against breast cancer both *in vitro* and *in vivo*. Moreover, IL-17E also directly acts on BC cells to induce apoptosis (118). Thus, the precise role of IL-17 members remains to be elucidated, their specific roles need to be considered from the aspects of both the tumor development stage and TME.

### 6.4 Lung cancer

IL-17 has been implicated in the promotion of lung cancer growth, in part by fostering a pro-inflammatory environment that leads to tumor cell proliferation, angiogenesis, and the recruitment of bone marrow-derived cells (122, 126). Increased IL-17 expression *in vivo* can alter lung cytokine secretion, T-cell function, and lung cancer development (126). IL-17A can promote the development of lung cancer by directly inhibiting the apoptosis through the ROS/Nrf2/p62 pathway leading to increased PD-L1 expression in NSCLC cells (119). Additionally, IL-17A promotes cell proliferation, migration, invasion, and metastasis, as well as angiogenesis, through the STAT3, NF-κB, JAK/STAT pathway, and CXCR2 (62, 120). Notably, IL-17A has been reported to mediate the migration, invasion, and dryness of NSCLC through the STAT3/NF-κB/Notch1 signaling (121). Meanwhile, IL-17A has been shown to up-regulate the expression of IL-8, MMP2, MMP9, and phosphorylated STAT3 (60, 123, 124), and ZEB1 to facilitate tumor invasion and migration (122, 123, 125). In addition, IL-17 can also up-regulate the expression of G-CSF through the NF-κB pathway, which recruits MDSCs to the TME and leads to resistance to PD-1 checkpoint blockade (126). Some studies have found that IL-17A promotes the secretion of CCL20 by cancerous cells, implicating the CCL20/CCR6/IL-17 axis as a potential new therapeutic target for lung cancer (127). IL-17A also stimulates the production of VEGF by cancer cells via the STAT3/GIV signaling pathway, promoting angiogenesis in non-small-cell lung cancer (61). Meanwhile, IL-17D promotes lung cancer progression by inducing TAMs infiltration via the p38 MAPK signaling pathway (53).

Recent studies have demonstrated that the interplay between IL-17 and lung microbiota can facilitate tumor initiation and progression. A recent clinical investigation revealed that commensal bacteria at the lung tumor site, resulting from adenovirus-mediated deletion of the tumor suppressor molecule P53, stimulated γδ T cells to secrete IL-17 (30). In the KP lung cancer model, dysbiosis of lower airway microbiota characterized by *Veillonella parvula* leads to Th17 cell recruitment, culminating in enhanced IL-17 production, which subsequently promotes lung tumor growth; a similar phenomenon has been observed in non-small cell lung cancer (NSCLC) (191).

However, it is important to note that some research has demonstrated that IL-17 may inhibit lung cancer progression under specific conditions. The absence of IL-17 has been shown to hinder the activation of the STAT1 pathway, resulting in accelerated tumor progression (128). Furthermore, IL-17 is essential in certain therapeutic contexts, such as treatments involving PI3K pathway inhibitors and Toll-like receptor agonists in Lewis lung carcinoma, where it can sustain the anti-tumor effects during lung transplantation tumors (129). Given our evolving understanding of IL-17’s role and mechanisms in lung cancer, anti-IL-17 therapy presents a promising novel therapeutic strategy for lung cancer patients.

### 6.5 Liver cancer

Liver cancer is closely associated with immune responses that involve IL-17A, which is highly expressed in hepatocytes (192). Clinical data showed that serum levels of IL-17 were significantly increased in CHC and HCC patients (193). At present, IL-17A has been used to study hepatitis, liver transplantation, liver cancer, cholangitis, and other liver diseases (194). Some studies have indicated that IL-17 in the Liver can stimulate progenitor cells (LPC) to promote their transformation into CSCs through miR-122 downregulation (130). Elevated IL-17A expression can aggravate liver damage by increasing lipid uptake and impairing cholesterol and fatty acid synthesis in hepatocytes (131). Concurrently, IL-17A activates the caspase 2-S1P-SREBP1/2 pathway, up-regulating the expression of DHCR7 and FASN, which subsequently promotes tumorigenesis (87).

IL-17A has been shown to promote the migration and invasion of HCC cells by increasing the expression of matrix metalloproteinases MMP2 and MMP9 through the NF-κB pathway and AKT2/STAT3 pathways (7, 132, 133). Literature reports that IL-17 may enhance the migration of neutrophils into HCC, where the neutrophils can stimulate the proangiogenic activity of tumor cells (134). Additionally, IL-17 activates the JAK2/STAT3 signaling pathway through the PI3K/AKT signaling pathway, leading to the production of IL-6 (132). Moreover, IL-17 can inhibit the anti-tumor effects of IFN-γ and up-regulate the expression of the negative feedback regulator PIAS1 by activating the JAK/STAT1 pathway, thereby accelerating HCC development (135). Studies have also found that IL-17 also can decrease the expression of cyclin D1 inhibiting tumor cell apoptosis process and promoting liver cancer (136). An independent study suggested that HSCs may participate in alcohol-induced HCC development through the IL-17-independent pathways or indirectly through the IL-17A-glutamate pathway (195).

However, IL-17A exerts a protective effect on the survival and function of HCC cells by inhibiting autophagic activity through the suppression of Bcl2 degradation (137). Meanwhile, IL-17F was shown to directly inhibit the vascular endothelial cells and decrease the expression of angiogenesis factors including IL-6, IL-8, and VEGF, thus inhibiting tumor angiogenesis and tumor growth (138). Therefore, further research is needed to elucidate the specific mechanisms by which HCC and IL-17A interact.

### 6.6 Prostate cancer

Prostate cancer (PCa) is another malignancy where IL-17 plays a multifaceted role. Recent studies have reported elevated levels of IL-17A, IL-17F, and IL-17RC in PCa patients (104). IL-17 has been shown to promote prostate cancer progression, primarily through the induction of EMT by enhancing MMP7 expression (139, 140). IL-17 could also up-regulate the expression of PD-L1 in LNCaP cells by the NF-κB and ERK1/2 signaling (141). Up-regulation of IL-17 expression also increases CTSK and the IL-17/CTSK/EMT axis controls the growth and spread of tumors in castration-resistant prostate cancer (CRPC), indicating a potential treatment target for therapy (142). It has also been found that IL-17 increases the expression of VCAM-1 in vascular endothelial cells through CD44-VCAM-1 interaction, thereby enhancing the adhesion of PCa cells to vascular endothelial cells to promote prostate cancer metastasis (143). Recent literature suggests that IL-17F activates the PI3K/AKT signaling pathway, promoting prostate cancer cell viability, proliferation, migration, invasion, and stemness *in vivo (* 148). Meanwhile, the IL-17 signaling pathway may have a significant impact on how immune cell infiltration and tumor glycolysis interact and findings demonstrate that the IL-17 induces the infiltration of MDSCs to promote prostate tumor growth (144–146, 196, 197). IL-17 also can activate the COX-2-VEGF pathway and plays a role in prostatic angiogenesis (147).

However, the formation of these vessels also provides a route for anti-tumor immune cells provides a way to reach the tumor site (4). At present, there are few studies on the specific mechanisms linking IL-17 and PCa is limited. A greater understanding of these mechanisms could offer additional treatment options for PCa patients.

### 6.7 Ovarian cancer

It has been found that endogenous IL-17A can promote the growth and metastasis of OvCa by increasing the expression of FABP4 and p-STAT3 (103). IL-17 also promotes the self-renewal of CD133 cancer stem-like cells (CSLCs) through NF-κB and p38 MAPK signaling pathways and facilitates ovarian cancer malignancy (149). IL-17 also induces the expression of programmed cell death 1 ligand (PD-L1) and its related factors (IL-6 and phosphorylated STAT3) (150). The presence of IL-17 in the TME also leads to an increase in TNF-α, which recruits myeloid cells through TNF receptor 1 (TNFR1) proinflammatory cytokines, further promoting tumor development (151). IL-17 has also been found to up-regulate metastasis-associated 1MTA1 mRNA and protein expression to promote ovarian cancer (OC) migration and invasion (152). On the one hand, IL-17 can also promote tumor growth by recruitment of numerous myeloid‐cell‐targeting cytokines and chemokines, these mediators promote the recruitment of immune cells and their pro‐tumorigenic programming in the OC TME (153). On the other hand, IL-17 could also promote angiogenesis and inhibit CXCL9 and CXCL10 production in ovarian tumor cells (8).

However, several studies have demonstrated that IL-17 can suppress the progression of ovarian cancer by recruiting CD4^+^T and CD8^+^T cells to exert an anti-tumor effect. Additionally, CXCL9 and CXCL10 can induce effector cells such as CD8^+^T and NK cells to mediate anti-tumor immunity (7). A more comprehensive understanding of the role of IL-17 in regulating ovarian cancer is needed to advance the treatment strategies for this disease, and further research is required to explore their interrelationship.

### 6.8 Gastric cancer

In gastric cancer, IL17 cells may promote tumor progression through IL17, IL22 and IL26 signaling or Tc17 (IL-17CD8 T cells) differentiation into exhausted T cells (154). In gastric cancer, up-regulation of IL-17 activates STAT3 signaling to promote tumor burden and metastasis (155). Application of AGS cells and mouse models showed that the IL-17A-activated STAT3 signaling pathway also induces up-regulation of VEGF-A expression and neovascularization, thereby promoting tumorigenesis (156). IL-17A produced by neutrophils exacerbates tumor growth by inducing CXC chemokines (CXCL1, CXCL2, CXCL3, CXCL5, CXCL8, and CXCL11) in gastric cancer cells to recruit neutrophils to the invasive edge. At the same time, peritumoral stromal neutrophils in turn promote invasive margin angiogenesis by up-regulating MMP9 expression and ultimately promote tumor progression (67, 157). At the same time, IL-17A secreted by cancer-associated neutrophils and cancer-associated fibroblasts (CAF) induces the expression of human antigen R (HuR) by activating the JAK2/STAT3 signaling pathway, which recognizes the mRNA encoding Snail and induces its translation. Thus, it promotes EMT, migration and invasion of gastric cancer cells, and promotes the transformation of quiescent gastric CSCs into invasive gastric CSCs (155). Secondly, IL-17A also activates the NF-κB/NADPH oxidase 1 (NOX1) signaling pathway by binding to IL-17RA and IL-17RC, and promotes the cell cycle transition from G1 to S phase, thereby promoting the proliferation of gastric cancer cells (80). However, another clinical study found that IL17A mRNA expression and intratumoral IL17A+ cell infiltration were correlated with anti-tumor immunotexture, and IL17A+ cell infiltration could be used as an independent prognostic biomarker for OS in gastric cancer, but further prospective validation is needed (198).

### 6.9 Pancreatic ductal carcinoma

Increased circulating Th17 cells and serum IL-17A were found to be involved in the development and metastasis of pancreatic ductal carcinoma (PDAC) in a clinical trial (199). It has been reported that IL17 plays a carcinogenic role in PDAC by recruiting neutrophils, triggering neutrophil extracellular traps (NETs), and eliminating CD8 T cells from the tumor (158). IL-17 could also regulate cluster cell development and stem cell characteristics of pancreatic cancer cells by increasing the expression of DCLK1, POU2F3, ALDH1A1, and IL17RC (159). Meanwhile, microbial-dependent IL-17 signaling increases DUOX2 signaling in tumor cells and promotes tumor development (9). The IL-17 axis can also synergistically promote PanIN/PDAC by up-regulating Notch activity through the classical NF-κB pathway *in vitro (* 160). In pancreatic epithelial cells with a KrasG12D mutation, IL-17A can directly stimulate the pancreatitis mediator to regenerate islet-derived 3-β, leading to STAT3 activation, thereby promoting PDAC development (161). IL-17B/RB is also involved in the development of PDAC. It has been found that activation of IL-17B/RB on tumor cells can subsequently phosphorylate and activate ERK1/2, enhance the production of a series of chemokines, such as C-C motif chemokine ligands, and ultimately promote the recruitment of neutrophils, lymphocytes, and endothelial cells. Thus, assisting PDAC invasion and metastasis (162). In addition, activated IL-17B/RB pathway in pancreatic stellate cells could also enhance PDAC tumor cell metabolism and proliferation (91).

### 6.10 Esophageal squamous cell carcinoma

Although studies have shown that IL-17A can promote the migration and invasion of EAC cells (esophageal squamous cell carcinoma) through ROS/NF-κB/MMP-2/9 signaling pathway activation (163). However, IL-17-producing tumor-infiltrating cells in patients with esophageal squamous cell carcinoma (ESCC) may play a protective role in the tumor microenvironment and can be used as a prognostic marker for ESCC patients. Il-17-producing mast cells were found to be densely located in the muscularis propria layer in ESCC and are thought to mediate tumor-protective immunity through the recruitment of effector CTLS, M1 macrophages, NK cells, T cells, and dendritic cells to the tumor site, thus serving as a favorable prognostic factor (164, 165). Studies have shown that IL-17A can promote ESCC tumor cells to produce more chemokines CCL2, CCL20, and CXCL13, enhance B cell migration, or enhance IgG-mediated antibody and complement-mediated cytotoxicity of B cells to tumor cells to inhibit tumor development. IL-17A could also enhance the direct killing ability of B cells stimulated by IL-17A through enhanced expression of granzyme B and FasL (165). Meanwhile, IL-17 stimulated ESCC tumor cells to release more CXC chemokines CXCL2 and CXCL3, which are involved in neutrophil migration. In addition, IL-17 enhances the direct killing ability of neutrophils by enhancing the production of cytotoxic molecules, including reactive oxygen species (ROS), MPO, TNF-related apoptosis-inducing ligand (TRAIL), and IFN-γ (166).

### 6.11 Other cancers

IL-17 has also been found to promote B16 (melanoma) tumor growth by inducing IL-6 expression and activating Stat3 signaling (167). IL-17 can also promote tumor progression *in vivo* by promoting the terminal exhaustion of CD8 T cells (168). IL-17 also induces EPHA5 via TRAF2 to recruit PIAS2 and ELAVL1 to promote melanoma development (68). However, it has also been found that IL-17 may better control the development of melanoma in mice, and the progression of melanoma is accelerated in IL-17-deficient mice (200). Meanwhile, the expression profile of IL-17 in BRAFV600-mutated melanoma suggests that IL-17 can promote the activation of T cells and neutrophils, contributing to the clinical benefit of immune checkpoint therapy (169). Tumor immunogenicity may be a key factor in these opposing results.

Clinical trials have also found that IL-17 may be associated with poor prognosis in patients with head and neck cancer (HNC). HNC patients with a higher percentage of IL-17-expressing cells in peripheral blood have a significantly lower 5-year overall survival, and IL-17 seems to promote the proliferation of human oral squamous carcinoma cells by producing IL-6 and VEGF-A (170). In neuroblastoma (NB), IL-17-producing γδT cells may produce some specific factors, mainly IL-17A, which may play a pro-tumor role (171).

## 7 Targeting IL-17 signaling for cancer immunotherapy

Previous studies have shown that IL-17 plays a dual role in cancer growth and tumor elimination. On the one hand, IL-17 supports tumor growth in the early stage of tumor development and already-formed tumors by directly signaling to cancer cells and indirectly inducing immune-suppressive tumor environments. On the other hand, IL-17 produced by γδ T cells and Th17 cells enhances anti-tumor immunity (200). Given the important role of IL-17 in cancer, increasing research is being conducted to target IL-17 signals for cancer treatment. Among them, Tumor immunotherapy has greatly improved the efficacy of cancer treatment.

### 7.1 IL-17 in cancer therapy resistance

A burgeoning body of evidence implicates that IL-17 is involved in the therapeutic resistance observed across various cancers. In breast cancer, IL-17-mediated ERK activation and HER1 phosphorylation promote resistance to docetaxel-based chemotherapy and tyrosine kinase inhibition in breast cancer cells (201, 202). The IL-17-CXCR2 axis also facilitates the recruitment of neutrophils to the tumor sites, thus conferring breast cancer resistance to chemotherapy (203). In CRC, IL-17-induced copper uptake in an STEAP4-dependent manner contributes to the resistance to 5-FU-induced caspase-3 activation in human colon cancer (204). Moreover, IL-17 was found to promote the viability of colorectal cells treated with Cisplatin, whilst blocking IL-17 signaling resulted in cell apoptosis (205). This indicates that IL−17 promotes the development of cisplatin resistance in CRC. It has been also shown that IL-17 is responsible for inducing resistance to antitumor and anti-angiogenic effects of drugs blocking VEGF (206).

Recently, several studies have linked enhanced IL-17 levels with immunosuppression in the TME. The Th17 signature cytokines: IL-17A, IL-17F, and IL-17AF heterodimer mediate the resistance of non-Th17 effector CD4^+^ T cells to immune suppression to induce suppressive resistance (207). Supporting this, clinical research has shown that IL-17 implication in therapeutic resistance against checkpoint inhibitors in lung, colorectal, melanoma, and breast cancer patients (208–211). It has been demonstrated that IL-17A augments PD-L1 expression through the p65/NRF1/miR-15b-5p axis and promotes resistance (212). On the other hand, IL-17A induces ROS production and increases Nrf2 and p62 expression in NSCLC, resulting in reduced PD-L1 degradation and increased PD-L1 expression (119). Additionally, IL-17-induced translation of HIFα in cancer-associated fibroblasts drives resistance to anti-PD-L1 mediated tumor regression in cutaneous squamous cell carcinoma (60). Meanwhile, IL-17 has been observed to cause CD8 cytotoxic T cell (CTL) desensitization in a mouse model of PD-1 antibody-resistant lung cancer and to promote tumor sensitivity to IL-6 and neutrophil depletion in Kras lung tumors in IL-17 transgenic mice, both of which make PD-1 immune checkpoint therapy-resistant (126, 208). Taken together, these findings indicate that IL-17 plays an important role in chemotherapy resistance in different types of cancers.

### 7.2 Targeting IL-17 in cancer immunotherapy

In recent years, an increasing number of antibodies targeting IL-17 have been developed, among which secukinumab, ixekizumab (IL-17A inhibitors) and brodalumab (IL-17RA inhibitor) have been approved. Various other drugs targeting IL-17A, such as CNTO 6785, ABT 122, COV A322, and ALX-0761, have entered clinical trials for testing (67).

IL-17 is an attractive target for cancer immunotherapy. Targeting IL-17 or IL-17R may enhance anti-tumor immune activity. There is evidence that anti-IL-17 antibodies can enhance anti-VEGF therapy for colorectal cancer (213). Monoclonal antibodies against IL-17RB can block pancreatic cancer metastasis by silencing multiple chemokines and extending the lifespan of mice with pancreatic cancer (90, 91).

Furthermore, targeting IL-17 affects the function of other immune cells in the TME. IL-17R-deficient mice showed increased infiltration of CD8 T cells and decreased infiltration of MDSCs in liver cancer tissue (146). A study on the effects of anti-IL-17 monoclonal antibodies on freshly resected tissues from 50 breast cancer patients suggested that targeting IL-17 may enhance breast cancer anti-tumor immune activity by inhibiting the immune checkpoint PD-L1 and suppressing MDSCs (210). In esophageal squamous cell carcinoma (ESCC), IL-17 promotes humoral immunity mediated by B cells by inducing chemokines and enhances the tumor-killing ability of B cells indirectly and directly by stimulating the production of IgG and granzyme B (166).

However, the efficacy of IL-17-targeting therapy is not always as expected. Clinical trials of neutralizing antibodies targeting IL-17 and IL-17RA in patients with Crohn’s disease have shown that disease worsening has been observed in patients treated with sukinumab (anti-IL-17) (214) and increased serum C-reactive protein, an indicator of inflammation, has been observed in patients treated with brodalumab (anti-IL-17RA) (215). *In vivo* studies in mice found that colitis-related epithelial damage and intestinal leakage were exacerbated in the absence of IL-17 signaling (216–218). In a man with metastatic colon cancer, administration of anti-IL-17 cleared psoriasis associated with pembrolizumab (anti-PD1) therapy but led to cancer progression, suggesting that IL-17 may have an antitumor effect (219). An increased number of peripherally circulating IL-17^+^ Th17 cells is associated with better prognostic markers and improved patient survival (220). IL-17 expression also correlates with a greater number of cytotoxic IFN-γ^+^CD4^+^ and IFN-γ^+^CD8^+^ T cells in ovarian cancer (8).

### 7.3 Targeting IL-17 in immune checkpoint blockade

Immune checkpoint blockade (ICB) has emerged as a pivotal strategy in clinical cancer therapy, with PD-1/PD-L1 being among the most effective targets. Recent studies indicate that IL-17 plays an important role in resistance to immune checkpoint therapy, and targeting IL-17 may enhance the efficacy of ICB. Combining IL-17A neutralization with immune checkpoint inhibitors (ICIs) has been shown to not only maintain the anti-tumor efficacy of ICIs but also to inhibit PD-L1 expression in breast cancer cells (210). Another study indicated that targeting IL-17A in a mouse model of breast cancer inhibited PD-L1 expression. Combined treatment with anti-IL-17 and anti-PD-L1 antibodies enhanced anti-tumor effects, even resulting in tumor eradication (221). In microsatellite stable (MSS) CRC mouse models, IL-17A increased PD-L1 expression through the p65/NRF1/miR-15b-5p axis, weakening the response to anti-PD-1 therapy. Blocking IL-17A improved the efficacy of anti-PD-1 therapy in MSS CRC mouse models (212). Similarly, our recent study has shown that CARG-2020 delivers a single chain IL-12, shRNA against PD-L1, and an antagonist of IL-17RA, the combination therapy not only regresses tumor growth but also prolongs the survival of mice in a syngeneic ectopic model of colorectal cancer and hepatocellular carcinoma (222). It was also observed that the combined administration of IL-17A monoclonal antibodies and anti-PD-L1 monoclonal antibodies significantly increased therapeutic efficacy by reducing PD-L1 expression in NSCLC tumor tissue (119). Furthermore, in KRAS/p53 mutant lung tumors, the effect of anti-PD-1 therapy was more pronounced in IL-17C-deficient KRAS lung cancer mice than in wild-type mice, suggesting that blocking IL-17C may improve the response to anti-PD-L1 therapy in lung cancer patients (223). In addition to the PD-1/PD-L1 immune checkpoints, IL-17 is also involved in anti-TIM-3 and anti-CTLA-4 immunotherapies. A study in a breast cancer model demonstrated that IL-17A-producing γδ T cells induced resistance to anti-PD-1 and anti-TIM-3 immune therapy (224). Another study showed that using PI3K inhibitors and Toll-like receptor agonists for immunotherapy induced IFN-γ^+^IL-17^+^ multifunctional T cells, mediating tumor immune rejection in mice (225). In BRAF V600-mutated melanomas, high levels of IL-17 gene expression supported the anti-tumor effects of dual ICI with anti-PD-L1 and anti-CTLA-4, but not with single anti-CTLA-4 or anti-PD-1 therapy (169).

Currently, there is a relative scarcity of clinical studies on the combination of anti-IL-17 antibodies with anti-PD-1 antibodies for cancer treatment. In a study conducted by SHUN Li et al., camrelizumab was used in combination with secukinumab to treat patients with advanced MSS CRC who had failed first or second-line treatments. Among them, one patient’s tumor profile showed MSS and low TMB (4.69 Muts/MB). High expression of IL-17A was also detected in the tumor tissue. After receiving the combined treatment of this regimen, a significant anti-tumor effect was observed (211). Another recent study analyzed the gene expression profiles and overall survival rates of melanoma patients with different genotypes treated with ICI and found that an increase in IL-17 GES in melanoma at the baseline of treatment was associated with an improved anti-tumor response to dual ICI therapy, but not related to monotherapy with anti-PD-1 or anti-CTLA-4 ICI (226). Additionally, there are clinical studies on the combination of anti-IL-17 antibodies and anti-PD-1 antibodies, such as CPDR001X2103 NCT02900664, which uses PDR001 (PD-1 inhibitor) in combination with CJM112 (anti-IL-17A antibody), canakinumab, and trametinib to treat advanced CRC, triple-negative breast cancer, and non-small cell lung cancer to verify their effects. However, the available data is insufficient.

### 7.4 Targeting IL-17 in radiation oncology therapy

Meanwhile, we found that targeting IL-17 is helpful for radiation oncology therapy (RT). Both mouse models and clinical data show that IL-17 expression in lung tissue is increased after irradiation (10). It is well known that radiation induces IL-17 secretion by Foxp3 ^+^Treg cells in diffuse large B-cell lymphoma (DLBCL) TME via IL-6 (227). It is highly likely that secreted IL-17 interacts with and affects other signaling axes, further affecting inflammation or immune regulation in the TME. In targeted radiotherapy (tRT) for lung cancer, IL-17 induces TRT-induced toxicity through inflammation, such as local acute toxicity of RILT, pulmonary fibrosis, pneumonia, etc (10). A direct link between IL-17 and RILT, also known as RILI, was demonstrated by the lower incidence of fibrosis and pneumonia after radiation induction in IL-17-deficient mice (228). It has also been found that IL-17-driven inflammation is closely related to pulmonary fibrosis (229). However, it has also been found that IL-17 can protect the whole body from gamma radiation during radiotherapy (11). IL-17 may act as a biomarker for predicting the toxicity caused by tRT. Targeting it shows the potential to reduce the side effects of tRT and improve treatment outcomes.

## 8 Concluding remarks

The IL-17 signaling plays a vital part in the induction of inflammation. Here we have summarized the literature regarding the emerging role of IL-17 signaling mainly in cancer pathogenesis and therapy. It was proposed that IL-17 may support cancer development by promoting chronic inflammation. Indeed, increased IL-17 signature genes have been observed in several human malignancies including CRC, breast cancer, and liver cancer. During the early stage of CRC tumorigenesis, IL-17 signaling directly promotes the proliferation of transformed colon epithelial cells in the sporadic tumor mouse model. IL-17 also indirectly induces suppressive tumor environments by inhibiting cytotoxic CD8^+^ T recruitment to tumors. Additionally, IL-17 directly signals to Tregs and promotes their maturation and function which constitutes a negative feedback loop controlling cancer-promoting inflammation in CRC. Whereas, in the established subcutaneous transplantation tumor model, IL-17 also exhibits anti-tumor activities by enhancing NK cell and CTL cell activation and by recruiting neutrophil, NK cell, CD4^+^, and CD8^+^ T cell into the TME. IL-17 has also been recognized in correlation with gut microbiome and lung microbiome during cancer progression. The role of IL-17 signaling in cancer remains convoluted due to its role in regulating the gut microbiome.

Targeting IL-17 not only enhances the efficacy of chemotherapy but also reduces the toxicity of radiotherapy. Increased IL-17 levels during radiotherapy cause a strong inflammatory response in the TME, which in turn causes serious short - and long-term adverse consequences, such as secondary cancers, and may adversely affect morbidity, mortality, and quality of life. However, IL-17 can protect the body from gamma radiation during radiotherapy, indicating that the protective or harmful response of IL-17 expression to radiation depends on the tissue type, microenvironment, and triggering factors. More studies are needed to determine the exact role of tissue-specific IL-17 sources in TRT-induced cancer development and toxicity.

The positive and negative effects of IL-17 in tumors may be caused by various factors. Firstly, the expression levels of different cytokines/receptors are different in different tumors. Secondly, it is also related to the stage of tumor development. The tumor microenvironment in the early and late stages of the tumor is different, and the effect of IL-17 may be different. At the same time, recent literature has found that the microbiome also has a regulatory effect on the secretion of IL-17. The ability of the microbiome is complex and variable, and its differences will also affect the effect of IL-17. Secondly, the different research models may also be the reasons for the different effects of IL-17. There are differences between the transplanted tumor model and the primary model, and the mouse model is limited to simulate the occurrence of human tumors, and the different forms of research (protein or mRNA) may also cause different results. At the same time, the effects of IL-17 from different cell sources are also different. Finally, the specific role of IL-17 signaling when integrated with other environmental factors is largely unknown.

Further studies are required to elucidate the potential contribution of IL-17-mediated inflammatory response in various stages of tumor formation and progression. In addition, more research is still needed to explore the roles of different sources of IL-17 cytokines in tumor pathogenesis. Detailed mechanistic insights into the IL-17 network in tumors will help inform appropriate targeting agents for cancer. Emerging evidence suggests that effective blockade of IL-17 signaling activity has been potentially employed as the adjuvant treatment for cancer. More preclinical studies will focus on the time windows for therapeutic agents against cancer treatment.

## Glossary

IL-17: Interleukin-17
Th17: T helper 17
Tc17: Cytotoxic T cells
γδT: Gamma delta T cell
NKT: Natural killer T cells
NK cells: Natural killer cell
AS: Ankylosing spondylitis
TME: Tumor microenvironment
MMP: matrix metalloproteinases
TAK1: TGF-activated kinase 1
DC: Dendritic cells
TAM: Tumor-associated macrophage
MDSCs: Myeloid-derived suppressor cells
Tregs: Regulatory T cells
BC: Breast cancer
TAN: tumor-associated neutrophils
CTLs: Cytotoxic T lymphocyte
NSCLC: Non-small cell lung cancer
PsA: Psoriatic arthritis
AS: Ankylosing spondylitis
IBD: Inflammatory bowel disease
MS: Multiple sclerosis
SLE: Systemic lupus erythematosus
CAC: Colitis-associated colorectal cancer
GC: Gastric cancer
CRC: Colorectal cancer
HCC: Hepatocellular carcinoma
ALD: Alcoholic liver disease
PDAC: pancreatic ductal adenocarcinoma
PA: palmitic acid
FGF: Fibroblast growth factors
PCa: Prostate cancer
PD-L1: Programmed cell death 1 ligand
OC: Ovarian cancer
ICB: Immune checkpoint blockade
ICI: Immune checkpoint inhibitors
MSS: Microsatellite stable
CSLCs: Cancer stem-like cells
LPC: Progenitor cell
ETBF: Bacteroides fragilis
G-CSF: Granulocyte colony-stimulating factor
C/EBP: CCAAT/enhancer binding protein
NF-κB: nuclear factor-κB
TNF: tumor necrosis factor
TRAF: TNF receptor associated factor
IKK: inhibitor of kappa B kinase
ERK: extracellular regulated protein kinases
JNK: c-Jun N-terminal kinase
AP-1: activator protein-1
GM-CSF: Granulocyte-Macrophage Colony Stimulating Factor
HFD: high fat diet
FABP: fatty acid-binding protein
STAT3: Signal Transducer and Activator of Transcription 3
NOX: nicotinamide adenine dinucleotide phosphate oxidases, LOH, loss-of heterozygosity
DHCR7: 7-dehydrocholesterol reductase
CRPC: castration-resistant prostate cancer
VCAM-1: vascular cell adhesion molecule 1
MTA1: Recombinant Metastasis Associated Protein 1
VEGF: vascular endothelial growth factor
ESCC: esophageal squamous cell carcinoma
RILT: radiation-induced lung toxicity
RILI: radiation-induced lung injury
tRT: targeted radiotherapy
EMT: epithelial-to-mesenchymal transition
GA: gastric cancer
CAF: cancer-associated fibroblasts
NOX1: NADPH oxidase 1
PDAC: pancreatic ductal carcinoma
NETs: neutrophil extracellular traps
ESCC: esophageal squamous cell carcinoma
ROS: reactive oxygen species
TRAIL: TNF-related apoptosis-inducing ligand
HNC: head and neck cancer
NB: neuroblastoma.
